# Cross-Phenotype Genomics Reveals Heterogeneous Genetic Susceptibility Architecture Relevant to Menopause

**DOI:** 10.3390/diagnostics16213705

**Published:** 2026-11-09

**Authors:** Gustavo Torres De Souza, Laura Vila-Vecilla, Valentina Russo, Caroline Brandão Chiovatto, Adil Yafou El Kayori

**Affiliations:** 1 Laboratory of Genetics, Fagron Genomics, Research and Development Department, 08226 Barcelona, Spain; 2 Fagron Brasil, São Paulo 04571-010, Brazil; caroline.chiovatto@fagrongenomics.com.br

**Keywords:** menopause, germline variants, cross-phenotype genomics, phenome-wide association study, fine-mapping

## Abstract

**Background**: Menopause is accompanied by substantial interindividual variation in hormonal, metabolic, cardiovascular, skeletal, and thrombotic phenotypes. Although the genetics of reproductive ageing and individual menopause-associated traits are increasingly characterised, the cross-phenotype architecture of individual menopause-relevant germline variants remains less well defined. **Methods**: We performed a variant-centred genomic analysis of 31 prespecified menopause-relevant germline variants. Cross-trait evidence comprised 766 variant-trait records across 52 phenotypes and was integrated with a targeted Pan-UK Biobank phenome-wide association study (PheWAS; 1462 tests across 57 endpoints), clinically relevant outcome analyses, linkage disequilibrium assessment, FinnGen R12 venous thromboembolism (VTE) associations, regional fine-mapping, and molecular annotation. Association strength, between-study heterogeneity, phenotypic breadth and sharing, and locus resolution were evaluated as distinct evidence dimensions. **Results**: In total, 25 of 31 variants showed at least one cross-trait association at *p* < 1 × 10^−5^ and 21 reached genome-wide significance. Phenotypic breadth varied markedly, from restricted profiles to 16 associated phenotypes, and strong associations frequently coexisted with substantial between-study heterogeneity. In Pan-UK Biobank, 222 associations involving 25 variants met false-discovery-rate correction, spanning hormonal, reproductive, cardiometabolic, skeletal, nutritional, hepatic, and neurological phenotypes. Cross-phenotype reconstruction identified recurrent connectivity through sex hormone-binding globulin, testosterone, adiposity, lipid, and skeletal traits, while linkage disequilibrium analyses distinguished phenotypic sharing from genetic redundancy. Clinically, *F5* rs6025 and *F2* rs1799963 showed strong VTE associations in FinnGen (OR 2.387 and 1.921, respectively), but markedly different locus resolution: rs6025 had posterior inclusion probability (PIP) 0.979 and constituted a single-variant 95% credible set, whereas rs1799963 had PIP 0.596 within a four-variant credible set. **Conclusions**: Menopause-relevant germline variation exhibits heterogeneous cross-phenotype architectures in which association strength, phenotypic breadth, between-study consistency, genetic independence, and locus resolution are not interchangeable. This variant-centred framework provides a basis for evaluating inherited susceptibility relevant to menopause and hormone exposure, while prospective individual-level studies remain necessary before translation into risk prediction or treatment selection.

## 1. Introduction

Menopause represents a major endocrine transition characterised by the cessation of ovarian reproductive function and substantial changes in the production and circulating availability of sex steroids. The timing of this transition itself has a substantial genetic component, with genome-wide studies identifying loci involved in DNA-damage response, ovarian reserve, and reproductive ageing, and demonstrating genetic overlap between variation in normal age at natural menopause and early menopause [1,2,3]. The biological consequences accompanying the menopausal transition are nevertheless heterogeneous and extend beyond reproductive function. Longitudinal studies have demonstrated accelerated bone mineral density loss around the final menstrual period, with circulating oestradiol, follicle-stimulating hormone, and other sex-hormone measures associated with skeletal changes [4,5]. The transition is also accompanied by changes in body composition and lipid profiles, including increased fat mass, declining lean mass, and changes in circulating lipids, although these trajectories reflect interacting effects of reproductive ageing and chronological ageing rather than a uniform consequence of menopause itself [6,7,8,9]. At the molecular level, circulating sex-hormone phenotypes also show substantial inherited variation: genome-wide studies in postmenopausal women have identified genetic determinants of sex hormone-binding globulin (SHBG) and signals related to circulating oestradiol and testosterone, while larger population studies have demonstrated sex-specific genetic architectures of testosterone and related hormone traits [10,11,12]. Together, these observations position menopause within a broader background of interindividual endocrine, metabolic, cardiovascular, and skeletal variation rather than as an isolated reproductive event.

This heterogeneity is clinically relevant because women enter and progress through menopause with different underlying susceptibility to conditions that become particularly important in midlife and later life. Cardiovascular risk factors change across the menopausal transition, supporting increased attention to cardiovascular prevention during this period [8], while bone loss around the final menstrual period contributes to the subsequent burden of osteoporosis and fracture [4,5]. Large-scale genomic studies further demonstrate extensive inherited architecture underlying both osteoporosis and venous thromboembolism (VTE), illustrating that clinically important postmenopausal phenotypes arise against a pre-existing background of genetic susceptibility [13,14]. Menopausal hormone therapy (MHT) provides an important clinical context in which such baseline heterogeneity becomes particularly relevant. MHT remains the most effective treatment for vasomotor symptoms and has established benefits for the prevention of bone loss, but its benefit–risk profile varies according to age, time since menopause, treatment formulation, route of administration, duration, and individual clinical characteristics [9,15,16]. Randomised evidence from the Women’s Health Initiative demonstrated that hormone therapy produces an outcome-specific balance of benefits and risks rather than a uniform treatment effect, with differences according to regimen and age at initiation [17,18]. The importance of timing is further supported by the ELITE trial, in which oral oestradiol reduced progression of carotid intima-media thickness when initiated within six years after menopause but not when initiated ten or more years after menopause, whereas KEEPS found no significant effect of either low-dose oral conjugated oestrogens or transdermal 17β-oestradiol on carotid atherosclerosis progression in recently menopausal women [19,20]. Route and formulation are similarly relevant to thrombotic risk: observational and meta-analytic evidence consistently associates oral oestrogen with greater VTE risk than transdermal administration, while the accompanying progestogen may also influence this relationship [21,22,23]. Importantly for inherited susceptibility, factor V Leiden and the prothrombin G20210A variant have been associated with substantially greater VTE risk in women exposed to hormone therapy, providing a well-established example in which germline background and an exogenous hormonal exposure may jointly influence clinical risk [24,25].

Genetic evidence relevant to menopause has, however, largely developed through separate phenotype-centred lines of investigation. Large-scale studies have characterised the genetic architecture of reproductive ageing [1,2,3], circulating sex hormones and SHBG [10,11,12], skeletal phenotypes [13], and thrombotic disease [14]. Genetic analyses have also begun to demonstrate that reproductive ageing itself relates to a considerably broader phenotypic landscape. A Mendelian-randomisation phenome-wide association study of genetically proxied age at natural menopause interrogated 18,961 health-related traits and identified associations extending beyond reproductive phenotypes to bone, cardiometabolic, hepatic, haematological, and cancer-related traits [26]. Complementary Mendelian-randomisation analyses in postmenopausal women have provided further evidence linking age at natural menopause with selected ageing-associated outcomes, particularly osteoporosis and fracture, while also illustrating that associations with menopause timing cannot be assumed to represent causal effects across all later-life diseases [27]. These studies establish the value of cross-phenotype genetic investigation, but address principally the phenotypic consequences of genetically determined reproductive ageing. A complementary question remains insufficiently characterised: how individual menopause-relevant germline variants distribute their associations across reproductive, hormonal, and clinically relevant phenotypes, and whether variants with strong associations necessarily show comparable phenotypic breadth, between-study consistency, or locus resolution. This distinction is important because statistical association strength, pleiotropic breadth, heterogeneity across contributing studies, linkage disequilibrium, and the degree to which an association resolves to a specific variant or locus represent related but non-equivalent dimensions of genetic evidence.

In the present study, we therefore investigated the cross-phenotype genetic susceptibility architecture of 31 prespecified menopause-relevant germline variants using complementary genomic evidence. We integrated cross-trait genome-wide association and meta-analytic evidence with a population-scale Pan-UK Biobank phenome-wide association analysis, a prespecified analysis of clinically relevant outcomes, linkage-disequilibrium assessment, disease-specific FinnGen evidence for VTE, regional fine-mapping, and locus-level molecular annotation. The analysis was designed to characterise differences in association strength, between-study heterogeneity, and phenotypic breadth; identify shared and distinct relationships across reproductive, hormonal, cardiometabolic, and other clinically relevant phenotypes; and examine whether selected strong clinical associations differ in their statistical and biological locus resolution. By integrating these dimensions rather than treating association significance alone as evidence of equivalent biological or clinical meaning, we aimed to define the heterogeneous cross-phenotype architecture of germline susceptibility relevant to menopause. The study evaluates genetic susceptibility architecture and does not assess individual-level risk prediction or genotype-specific response to MHT.

## 2. Methods

### 2.1. Study Design, Variant Set, and Analytical Resources

We performed a variant-centred, cross-phenotype genomic analysis of 31 prespecified menopause-relevant germline variants using complementary summary-statistic, linkage disequilibrium (LD), fine-mapping, and molecular annotation resources. The variants were selected before the present analysis on the basis of previously established biological and literature evidence related to reproductive ageing, sex-hormone biology, and menopause-relevant clinical phenotypes. Variant inclusion therefore preceded the cross-phenotype analyses reported here and was not determined by the statistical results obtained in this study. Variant identifiers, genomic positions, alleles, associated genes/loci, and original biological rationale are provided in Table 1. The study used exclusively previously published or publicly available summary-level data, with no recruitment of participants or access to identifiable individual-level data.

The primary cross-trait evidence layer was derived from an 81-trait GWAS evidence database and comprised 766 retained variant-trait records across the 31 variants and 52 menopause-relevant traits. Available statistics included effect estimates (β or odds ratio [OR]), standard errors (SEs), fixed- and random-effects estimates, association *p* values, between-study heterogeneity (I^2^), contributing-study counts, and reported sample sizes. Population-scale phenotypic associations were examined using Pan-UK Biobank summary statistics [28], yielding 1462 exact-variant tests across 57 endpoints for 28 represented variants, including a prespecified clinical subset of 408 tests across 16 outcomes. Disease-specific thrombotic evidence was obtained from FinnGen Release 12 [29], including venous thromboembolism (VTE) summary statistics and ±1 Mb regional data surrounding *F5* and *F2*. LD was evaluated using European-ancestry UK Biobank whole-genome LD matrices [30] and targeted LDlink analyses based on the 1000 Genomes Project reference populations [31,32]. These statistical layers were complemented by individually traceable locus-level molecular and functional annotations, as described below.

Across resources, variants were harmonised by rsID and genomic position, using GRCh37 coordinates where required by the source dataset. Effect and reference alleles, effect estimates, SEs, association *p* values, phenotype type, and available sample-size or case–control information were retained rather than collapsed across resources. For Pan-UK Biobank, rsID, position, and allele matching were audited against the source data, with the alternate allele retained as the reported effect allele. Numerical *p* values recorded as zero because of floating-point underflow were treated as values below computational precision rather than true zero. Sample sizes were not summed across traits or evidence sources because contributing cohorts may overlap. Accordingly, the analytical resources were treated as complementary evidence layers for cross-phenotype and locus-level interpretation and were not assumed to constitute statistically independent replication datasets.

### 2.2. Cross-Trait Association Strength, Between-Study Heterogeneity, and Phenotypic Breadth

Cross-trait association patterns were evaluated using 766 variant-trait records representing 31 variants and 52 unique phenotypes. For each variant-trait association, fixed-effect estimates, standard errors, *p* values, effect direction, contributing-study and sample-size information, and random-effects estimates where available were retained. Associations were summarised at three prespecified thresholds: *p* < 0.05 for descriptive purposes, *p* < 1 × 10^−5^ as a descriptive threshold for strong association signals, and *p* < 5 × 10^−8^ as the conventional threshold for genome-wide significance [33]. The *p* < 1 × 10^−5^ threshold was not interpreted as a study-specific multiple-testing correction.

Between-study consistency was evaluated separately from association strength using I^2^, where at least two contributing studies were available; I^2^ estimates the proportion of observed variation attributable to between-study heterogeneity rather than sampling error [34,35]. Fixed- and random-effects results were retained separately and were not combined into a composite evidence measure. Phenotypic breadth was defined, for each variant and statistical threshold, as the number of unique associated phenotypes, thereby preventing repeated records for the same phenotype from inflating breadth. Where phenotypes were subsequently organised into broader biological or clinical domains, domain breadth was considered separately from phenotype breadth. No composite score or variant classification was derived from association strength, heterogeneity, or phenotypic breadth.

### 2.3. Population-Scale Targeted PheWAS and Clinically Relevant Outcomes

A targeted variant-centred PheWAS was performed using precomputed Pan-UK Biobank summary statistics [28], following the general PheWAS framework of testing genetic variants across multiple phenotypes [36]. Exact-variant data were available for 28 of the 31 study variants, yielding 1462 tests across 57 endpoints. CYP19A1 rs10046, *F5* rs6025, and SERPINE1 rs1799762 lacked eligible exact-variant results in the extracted Pan-UK Biobank dataset. Original phenotype definitions were retained, with endpoints organised into seven predefined families: cardiometabolic (566 tests), folate/nutrition (248), primary hormone/reproductive (208), neurological/ageing (156), bone/musculoskeletal (152), safety/pleiotropy (80), and symptoms/treatment proxies (52). Multiple testing was addressed across all 1462 variant–endpoint tests using the Benjamini–Hochberg false discovery rate (FDR) procedure [37] and Bonferroni correction (*p* < 0.05/1462). Endpoint families were retained for phenotype organisation and reporting rather than as separate multiple-testing correction families.

A prespecified clinical subset comprised 408 tests across 16 outcomes selected from the targeted PheWAS according to their direct clinical relevance to menopause-associated cardiovascular, metabolic, hepatic, and reproductive health. For binary outcomes, β estimates were converted to odds ratios as OR = exp(β), with 95% confidence intervals calculated as exp(β ± 1.96SE); quantitative-trait effects were retained on their original β scale. For each variant, the statistically strongest association was defined by the minimum valid *p* value and was distinguished from associations selected a priori for biological or clinical relevance. Marginal Pan-UKBB genotype–phenotype associations were not interpreted as treatment-response or genotype-by-treatment effects.

### 2.4. Reconstruction of Cross-Phenotype Genetic Architecture

Cross-phenotype architecture was defined using a bipartite network in which germline variants and phenotypes constituted separate node classes and statistically supported variant-phenotype associations formed edges, consistent with genotype–phenotype network approaches for investigating shared genetic architecture and pleiotropy [38,39]. Local and Pan-UKBB associations were retained as distinct evidence layers rather than pooled by *p* value. Edges preserved association source, phenotype, effect direction and magnitude, statistical significance, and LD information. Network characteristics were prespecified to include variant and phenotype degree, unique phenotype breadth, shared phenotypes between variants, and cross-domain connectivity.

Same-locus variants were annotated using pairwise LD to distinguish correlated associations from convergence across genetically distinct signals. Observed phenotype sharing was evaluated from the reconstructed variant-phenotype edge sets while preserving variant and phenotype connectivity information. Network-derived measures were interpreted as patterns of cross-phenotype association rather than evidence of epistasis or causality. The previously generated all-pair Jaccard matrix was retained for provenance only and was not used for network inference.

Statistically supported phenotypes were additionally grouped into seven biologically defined domains: hormonal/reproductive, cardiovascular/thrombotic, metabolic/adiposity, skeletal/musculoskeletal, nutritional/one-carbon metabolism, neurological/ageing, and hepatic/other. Domain breadth was defined as the number of distinct domains containing at least one supported association per variant, separately for local associations (*p* < 1 × 10^−5^) and Pan-UK Biobank associations (BH-FDR q < 0.05). Phenotype sharing was further evaluated using 2000 degree-preserving bipartite randomisations per evidence layer, implemented through double-edge swaps preserving variant and phenotype degrees. Sharing statistics were calculated between variants assigned to different genes and compared with their randomised distributions using empirical upper-tail *p* values. Domain breadth was interpreted as a descriptive measure rather than evidence of statistically independent phenotypes, while permutation testing evaluated sharing conditional on observed connectivity.

### 2.5. Linkage Disequilibrium, VTE Association, and Regional Fine-Mapping

LD between selected same-locus variants was characterised using the UK Biobank European whole-genome LD resource covering up to approximately 18 million variants [30], supplemented by targeted LDlink analyses using the European 1000 Genomes Project reference population [31,32]. Pairwise r and r^2^ were used to assess correlation between variants; D’ was retained only for targeted LDlink comparisons. These estimates were used to distinguish correlated markers from approximately independent genetic signals.

VTE associations for *F5* rs6025 (Factor V Leiden) and *F2* rs1799963 (G20210A) were evaluated using FinnGen Release 12 endpoint I9_VTE, comprising 26,333 cases and 474,015 controls (*N* = 500,348) [29]. Effect allele, log-OR (β), SE, allele frequency, and *p* value were extracted directly from the summary statistics, with ORs and 95% confidence intervals calculated from β and SE. Regional association data were additionally extracted within ±1 Mb of each locus. The resulting *F5* region contained 15,644 variants, of which 15,172 had resolved rsIDs, and the *F2* region contained 11,358 variants, of which 10,935 had resolved rsIDs; this reduction primarily reflected rsID availability rather than association-based filtering.

Regional statistical prioritisation was performed using Wakefield approximate Bayes factors (ABFs) calculated from β and SE [40]. Normal priors on the log-OR were evaluated at standard deviations of 0.10, 0.20, and 0.30. Within each region, ABFs were normalised to obtain variant posterior probabilities under a single-causal-signal model, variants were ranked by posterior probability, and 95% credible sets were constructed by cumulative posterior probability. Anchor-variant rank and posterior probability were retained for each locus. These analyses were interpreted as statistical fine-mapping and prioritisation and not as evidence of molecular causality or independence among variants within a credible set.

### 2.6. Molecular and Locus-Level Interpretation

Molecular interpretation was performed for statistically supported and biologically informative loci using complementary, source-traceable annotations. Variant consequence and genomic context were evaluated using Ensembl Variant Effect Predictor annotations [41], while positional gene relationships were recorded separately from molecular QTL evidence. Tissue-specific eQTL evidence was evaluated where available, including GTEx data [42]. LD relationships, fine-mapping rank, credible-set membership, and locus-specific primary literature were integrated as additional parallel evidence types. Positional, coding, regulatory, and statistical locus evidence were not treated as equivalent or combined into a composite evidence score. Physical overlap with an eQTL was considered regulatory context and was not interpreted as statistical colocalisation in the absence of a formal colocalisation analysis.

Detailed interpretation focused on loci showing strong clinical or hormone/reproductive associations, cross-phenotype relevance, or informative locus-resolution evidence, while additional variant-level annotations and analytical data were retained by the authors and may be made available upon reasonable request. Previously generated composite pathway, tissue, pharmacogenetic, convergence, evidence-grade, and biological-module fields were excluded from the present analysis.

All statistical analyses were two-sided unless otherwise specified by the source dataset. Effect estimates were retained on their appropriate quantitative or log-odds scales, 95% confidence intervals were reported where applicable, and multiple-testing procedures were applied within their predefined analysis families. Numerically underflowed *p* values were not interpreted as zero, unavailable statistics were not imputed, and sample sizes were not aggregated across potentially overlapping datasets. Version-controlled analytical scripts and intermediate outputs were retained for reproducibility. No composite genetic score, variant-tiering system, individual-level risk model, treatment-response model, or genotype-by-MHT interaction model was constructed.

## 3. Results

### 3.1. Menopause-Relevant Germline Variants Show Heterogeneous Cross-Phenotype Association Profiles

The local cross-trait analysis comprised 766 variant-trait records across 31 germline variants and 52 menopause-relevant phenotypes. Twenty-five of the 31 variants (80.6%) showed at least one association at *p* < 1 × 10^−5^, and 21 (67.7%) reached genome-wide significance (*p* < 5 × 10^−8^). The extent of association differed markedly between variants. At *p* < 1 × 10^−5^, the number of associated phenotypes per variant ranged from 1 to 16 (median, 3), while genome-wide-significant associations ranged from 1 to 15 phenotypes among the 21 variants reaching this threshold (median, 3).

The resulting profiles ranged from relatively focused associations to broad cross-phenotype patterns. *F5* rs6025 showed its strongest association with VTE (β = 0.87009, *p* = 5.91 × 10^−250^) and was associated with three phenotypes at *p* < 1 × 10^−5^. In contrast, the two *APOE* variants showed the greatest phenotypic breadth, with 16 significant phenotypes for rs429358 and 13 for rs7412 at the same threshold. Prominent hormone-related signals were observed for *SHBG*, *CYP3A7*, and *UGT2B15*, including associations with *SHBG* and testosterone, while *ESR1*, *ESR2*, and *CYP19A1* contributed reproductive, hormonal, and skeletal associations. One-carbon metabolism loci showed more restricted profiles, with significant associations involving folate, platelet count, and selected metabolic or treatment-related phenotypes. Collectively, these results demonstrate substantial variation in both the breadth and phenotypic composition of association profiles across the prespecified variant set, rather than a uniform pattern of menopause-related genetic susceptibility (Figure 1).

### 3.2. Association Strength, Consistency, and Phenotypic Breadth Represent Distinct Properties of Genetic Evidence

Association strength varied independently of between-study consistency. Between-study heterogeneity was evaluated among associations supported by at least two contributing studies. Of the 80 multi-study associations reaching *p* < 1 × 10^−5^, I^2^ ranged from 0% to approximately 100% (median, 34.4%; IQR, 0–94.7%), with 38/80 (47.5%) showing I^2^ > 50% and 32/80 (40.0%) showing I^2^ > 75%. Among the 66 multi-study associations reaching genome-wide significance, 32/66 (48.5%) had I^2^ > 50% and 28/66 (42.4%) had I^2^ > 75% (median I^2^, 45.6%; IQR, 0–96.5%). Thus, substantial between-study heterogeneity was present in a considerable proportion of the strongest multi-study associations.

Contrasting individual associations illustrated this separation. *F5* rs6025 showed an exceptionally strong association with VTE (β = 0.87009, *p* = 5.91 × 10^−250^); however, this estimate was derived from a single contributing study and therefore did not provide an informative assessment of between-study heterogeneity. Conversely, *CYP3A7* rs45446698 showed a very strong fixed-effect association with testosterone (β = 0.10358, *p* below numerical precision) despite I^2^ = 99.73%, while *UGT2B15* rs1902023 was associated with *SHBG* (β = −0.01052, *p* = 5.91 × 10^−68^) with I^2^ = 99.27%. Similarly, the *SHBG* rs727428 association with *SHBG* (β = −0.07894, *p* below numerical precision) showed I^2^ = 99.99%. The corresponding random-effects *p* values for these three highly heterogeneous associations were 0.0466, 0.907, and 0.846, respectively, further illustrating the sensitivity of inference to between-study variability.

A similar issue was evident for *F2* rs1799963, for which the grouped local VTE evidence produced a significant fixed-effect estimate (*p* = 9.25 × 10^−20^) but extreme heterogeneity (I^2^ = 99.54%) and a non-significant random-effects result (*p* = 0.614). Phenotype-specific VTE evidence is therefore considered separately in Section 3.5. Together with the differences in phenotype breadth observed in Section 3.1, these findings show that statistical significance, between-study consistency, and cross-phenotype breadth capture distinct properties of the genetic evidence (Figure 2).

### 3.3. Targeted PheWAS Identifies Hormonal, Reproductive, Cardiometabolic, and Clinical Associations

The targeted Pan-UK Biobank PheWAS comprised 1462 exact-variant tests across 57 endpoints and 28 represented variants. After multiple-testing correction across all 1462 variant–endpoint tests, 222 associations involving 25 variants met the Benjamini–Hochberg false discovery rate (FDR) threshold, and 116 associations involving 22 variants remained significant after Bonferroni correction. Significant associations extended across hormonal and reproductive phenotypes as well as cardiometabolic, skeletal, nutritional, hepatic, and neurological endpoints, indicating that the cross-phenotype patterns observed in the local evidence were also evident at population scale.

Within the prespecified clinical subset, 408 tests across 16 outcomes yielded 14 FDR-supported associations involving nine variants and seven endpoints, of which seven remained Bonferroni significant. The strongest clinical associations involved *APOE*: rs429358 was associated with increased odds of coronary atherosclerosis (OR = 1.138, 95% CI 1.107–1.170; *p* = 1.58 × 10^−20^) and myocardial infarction (OR = 1.139, 95% CI 1.102–1.177; *p* = 1.15 × 10^−14^), whereas rs7412 showed associations in the opposite direction for coronary atherosclerosis (OR = 0.829, 95% CI 0.799–0.860; *p* = 2.24 × 10^−23^) and myocardial infarction (OR = 0.858, 95% CI 0.821–0.897; *p* = 9.12 × 10^−12^). Additional FDR-supported associations included *ESR2* rs4986938 with atrial fibrillation (OR = 0.946, 95% CI 0.925–0.967; *p* = 8.45 × 10^−7^), *SHBG* rs1799941 with atrial fibrillation (OR = 0.958, 95% CI 0.935–0.982; *p* = 7.53 × 10^−4^), and *SHBG* rs727428 with type 2 diabetes (OR = 0.968, 95% CI 0.949–0.987; *p* = 1.20 × 10^−3^).

Reproductive associations were observed for *CYP1A1* rs4646903 and rs1048943 with menopausal/perimenopausal disorders (OR = 1.080 and 1.131, respectively; FDR q = 0.0218 and 0.0318), while *CYP17A1* rs743572 and *CYP3A7* rs45446698 were associated with the Pan-UKBB “Had menopause” endpoint (OR = 0.953 and 1.121; q = 0.0252 and 0.0327, respectively). The source phenotype comprised 145,380 endpoint-positive observations and 54,896 comparison participants and is retained here using its original Pan-UKBB terminology rather than interpreted as a direct measure of menopausal timing (Figure 3).

A notable cross-phenotype association was observed for *F2* rs1799963, for which the A allele was associated with lower circulating *SHBG* (β = −0.04438, SE = 0.01162; *p* = 1.33 × 10^−4^; q = 0.00146; *N* = 381,526). However, the strongest Pan-UKBB association for this variant was with triglycerides (β = −0.08775; *p* = 2.40 × 10^−13^; q = 8.16 × 10^−12^), indicating that the *SHBG* association represents one component of a broader cross-phenotype profile rather than its predominant non-thrombotic association. The *SHBG* finding therefore provides hormone-related context for *F2* but does not establish a mechanistic relationship between *SHBG* and thrombotic susceptibility.

### 3.4. Reconstructed Cross-Phenotype Architecture Distinguishes Shared Connectivity from Locus-Specific Associations

Reconstruction of the cross-phenotype architecture demonstrated marked differences in connectivity among the analysed variants. At *p* < 1 × 10^−5^, the local evidence layer contained 95 unique variant–phenotype associations involving 25 variants and 27 phenotypes. Connectivity ranged from single supported phenotypes to 16 phenotypes for *APOE* rs429358 and 13 for rs7412. Hormone-associated loci also showed substantial connectivity, including *SHBG* rs727428 (nine phenotypes), *CYP3A7* rs45446698, *SHBG* rs1799941, and *UGT2B15* rs1902023 (six each). Thus, statistically supported cross-phenotype breadth was concentrated in a subset of variants rather than distributed uniformly across the variant set.

The Pan-UKBB layer showed a similar but broader architecture, with 222 FDR-supported edges involving 25 variants and 44 phenotypes. *APOE* rs429358 and rs7412 again showed the greatest phenotype connectivity, with 23 and 19 associated endpoints, respectively, followed by *SHBG* rs1799941 (16), *SHBG* rs727428 and *CYP3A7* rs45446698 (15 each), and *SULT1A1* rs1042028 and *MTHFR* rs1801133 (14 each). Several of these variants also connected multiple endpoint families: both *APOE* variants, *CYP3A7* rs45446698, and *SHBG* rs727428 had FDR-supported associations spanning five Pan-UKBB phenotype families.

Grouping related phenotypes into broader biological domains reduced the apparent breadth of several highly connected variants. In the local analysis, APOE rs429358 and rs7412 showed associations with 16 and 13 phenotypes, respectively, distributed across four and three domains. In Pan-UK Biobank, the corresponding associations comprised 23 and 19 endpoints spanning six domains each. SHBG rs727428 showed nine local phenotypes across three domains and 15 Pan-UK Biobank endpoints across five domains, whereas SHBG rs1799941 showed six local phenotypes across four domains and 16 Pan-UK Biobank endpoints across five domains. These findings indicate that phenotype counts partly reflect multiple associations within related biological systems, although several variants retain substantial cross-domain breadth.

Phenotype sharing was particularly evident at the intersection of hormonal and cardiometabolic traits. In Pan-UKBB, SHBG was FDR-associated with 14 variants and testosterone with 11, while body-fat percentage, waist circumference, triglycerides, and platelet count were associated with 13, 12, 10, and 10 variants, respectively. The local evidence independently showed recurrent connectivity through testosterone (12 variants), BMD (11), SHBG (nine), and triglycerides (eight). At the local *p* < 1 × 10^−5^ threshold, for example, *APOE* rs429358 and *SHBG* rs727428 shared eight associated phenotypes, including SHBG, testosterone, triglycerides, lipid-related traits, BMI, and waist-to-hip ratio. *UGT2B15* rs1902023 and *SHBG* rs727428 shared six phenotypes spanning hormone and lipid traits. These relationships indicate that the observed architecture includes cross-locus phenotype sharing rather than being restricted to associations among variants within the same gene.

Degree-preserving permutation analyses did not demonstrate significant excess cross-gene phenotype sharing relative to the observed connectivity structure. In the local association matrix, 55 variant pairs shared at least two phenotypes, compared with a null mean of 53.46 (95% null interval, 45–62; empirical *p* = 0.397). Similarly, 22 pairs shared at least three phenotypes, compared with 21.09 expected (*p* = 0.434). In Pan-UK Biobank, 152 pairs shared at least two endpoints, compared with a null mean of 161.60 (95% null interval, 150–172; *p* = 0.960), while 57 pairs shared at least five endpoints, compared with 51.77 expected (*p* = 0.085). Thus, although extensive phenotype sharing was observed, the tested global measures did not demonstrate enrichment beyond expectations after accounting for variant and phenotype degrees.

Targeted LDlink analysis using the European 1000 Genomes reference population further distinguished shared phenotypic connectivity from genetic redundancy. The *ESR1* rs2234693/rs9340799 and *CYP19A1* rs749292/rs10046 pairs showed appreciable same-locus LD (r^2^ ≈ 0.61 and 0.58, respectively), whereas *APOE* rs429358/rs7412 showed minimal pairwise LD (r^2^ = 0.012). Consequently, the extensive and partly overlapping *APOE* phenotype profiles cannot be attributed simply to high LD between the two analysed variants. Overall, the reconstructed architecture identifies a combination of restricted signals, broadly connected variants, and shared hormone–metabolic phenotypes, while demonstrating that phenotypic convergence and locus correlation represent distinct features of the genetic architecture.

### 3.5. F5 and F2 Confer Strong VTE Susceptibility but Differ Markedly in Locus Resolution

Both thrombophilia variants showed strong associations with VTE in FinnGen R12. For *F5* rs6025, the T allele was associated with OR = 2.387 (95% CI 2.270–2.511; *p* = 5.90 × 10^−250^), with an effect-allele frequency of 0.01995. For *F2* rs1799963, the A allele was associated with OR = 1.921 (95% CI 1.734–2.128; *p* = 9.50 × 10^−36^), with an effect-allele frequency of 0.00509. These associations were derived from the same VTE endpoint comprising 26,333 cases and 474,015 controls.

Despite the strong disease associations at both loci, regional fine-mapping showed substantially different statistical resolution. Among 15,172 rsID-resolved variants surrounding *F5*, rs6025 ranked first with a posterior inclusion probability (PIP) of 0.9787 and alone constituted the 95% credible set at a prior log-OR SD of 0.20. In the *F2* region, rs1799963 also ranked first among 10,935 variants, but its PIP was 0.5959 and the 95% credible set contained four variants. The three additional *F2* credible-set variants were correlated with rs1799963 (r^2^ = 0.560, 0.658, and 0.737), indicating that they represent correlated candidate signals rather than independent VTE associations.

Fine-mapping sensitivity analyses supported this contrast. Across prior log-OR SDs of 0.10, 0.20, and 0.30, rs6025 remained the sole variant in the *F5* 95% credible set, with PIP values of 0.9783–0.9788. For *F2*, rs1799963 remained rank 1 at all three priors, but its PIP ranged from 0.5563 to 0.6026 and the credible set consistently contained four variants. Thus, the principal difference between these loci was not the presence of VTE susceptibility, which was strong for both variants, but the degree to which the regional association could be statistically resolved to the selected variant (Figure 4).

### 3.6. Locus-Level Evidence Reveals Unequal Functional Resolution Across Menopause-Relevant Associations

Integration of variant consequence, LD, fine-mapping, molecular annotation, and cross-phenotype associations revealed substantial differences in the level of functional resolution achieved across loci. *F5* rs6025 represented the most resolved example: the missense Factor V Leiden variant [43] combined a strong VTE association with a single-variant 95% credible set and PIP of 0.9787. In contrast, *F2* rs1799963 is a 3′-UTR variant [44] with similarly strong VTE susceptibility but lower fine-mapping resolution, remaining within a four-variant credible set with PIP = 0.5959. Its broader phenotype profile included triglycerides and SHBG, while the molecular layer provided only nominal liver eQTL support (NES = 0.468, *p* = 0.0186) and no corresponding whole-blood association (*p* = 0.270). In the absence of formal colocalisation, this expression signal was therefore retained as functional context rather than evidence of a shared causal mechanism.

Locus structure provided an additional distinction between apparent and independent cross-phenotype support. In the UK Biobank European LD reference, the two coding APOE variants rs429358 and rs7412 showed minimal pairwise LD (r^2^ = 0.0159) while displaying strong, directionally distinct cardiovascular and metabolic associations, indicating that their phenotype profiles should not be treated as redundant solely because they occur within the same gene. Conversely, the *ESR1* rs2234693/rs9340799 and *CYP19A1* rs10046/rs749292 pairs showed substantial LD (r^2^ = 0.645 and 0.632, respectively), indicating partly overlapping locus information. The two *SHBG* variants showed more modest correlation (r^2^ = 0.270), despite prominent hormone-related associations.

Functional plausibility was likewise not synonymous with statistical consistency. Several steroid- and hormone-related loci showed strong biomarker associations but substantial between-study heterogeneity, including *SHBG* rs727428 and *UGT2B15* rs1902023, for which the principal *SHBG* associations had I^2^ values of 99.99% and 99.27%, respectively. Taken together, these locus-level contrasts show that strong association does not necessarily imply cross-study consistency, genetic independence, fine-mapping resolution, or equivalent functional interpretability.

## 4. Discussion

The present study demonstrates that menopause-relevant germline variation is characterised by markedly heterogeneous cross-phenotype architectures rather than by a uniform pattern of genetic susceptibility. Across the 31 prespecified variants, association strength, phenotypic breadth, between-study consistency, clinical phenotype connectivity, LD independence, and locus resolution provided related but non-equivalent information. This distinction extends an already substantial literature establishing the genetic architecture of reproductive ageing and age at natural menopause (ANM) [2,3]. Previous studies have also connected ANM-associated genetic variation with broader health outcomes: Sarnowski et al. related genetically determined earlier menopause to cardiovascular events [45], Magnus et al. examined genetically predicted ANM across 18,961 phenotypes using an MR-PheWAS framework [26], and Lankester et al. investigated potential causal relationships between ANM and ageing-associated outcomes using Mendelian randomisation [27]. Accordingly, the novelty of the present analysis does not lie in demonstrating that menopause genetics extends beyond reproductive phenotypes. Rather, to our knowledge, it lies in applying a variant-centred framework that jointly characterises association strength, between-study heterogeneity, phenotypic breadth and sharing, clinically relevant associations, LD independence, and statistical locus resolution across prespecified menopause-relevant germline variation. This approach addresses a different question from phenotype-centred analyses of genetically predicted menopause timing and provides a framework for distinguishing different dimensions of genetic evidence that might otherwise be conflated.

This distinction was evident across the cross-trait architecture. Some variants showed relatively restricted profiles, whereas others were connected to numerous hormonal, metabolic, skeletal, cardiovascular, or other phenotypes. The two *APOE* variants represented the broadest profiles despite very low pairwise LD, whereas same-locus *ESR1* and *CYP19A1* variants showed appreciable LD, illustrating that multiple associations within a locus cannot automatically be considered independent evidence. Equally importantly, statistical strength did not imply consistency: several exceptionally strong hormone-related fixed-effect associations also displayed pronounced between-study heterogeneity. These findings are compatible with the broader complexity already observed for reproductive-ageing and sex-hormone genetics, in which multiple loci, allelic heterogeneity, sex-specific effects, and context-dependent genetic effects contribute to circulating hormone variation and reproductive phenotypes [3,11,12]. Haas et al., for example, demonstrated that the genetic architecture of circulating sex hormones differs according to menopausal status, including significant heterogeneity at *CYP3A7* for testosterone [46]. Thus, phenotypic sharing in the present study is most appropriately interpreted as evidence of cross-phenotype genetic connectivity rather than as proof of a single shared causal pathway. Similarly, high connectivity does not necessarily indicate greater biological importance than a strong but phenotype-specific association.

The domain-level analysis further demonstrated that some of the observed phenotypic breadth reflected multiple associations within related biological systems, although several variants retained associations across distinct domains. Moreover, degree-preserving permutation analyses did not demonstrate excess global phenotype sharing beyond expectations based on the observed connectivity structure. These findings reinforce the distinction between descriptive cross-phenotype connectivity and statistically enriched genetic architecture.

Hormonal and reproductive loci provided a particularly informative connection between menopause-related biology and broader metabolic and clinical phenotypes. Genetic regulation of circulating SHBG and sex steroids is well established, including associations at *SHBG*, *CYP3A7*, *UGT2B15*, and other loci represented or biologically related to those examined here [10,11,12,46]. Prescott et al. identified rs727428 as a genome-wide significant determinant of SHBG in postmenopausal women [10], while Coviello et al. demonstrated broader allelic and sex-dependent heterogeneity in the genetic regulation of SHBG [11]. These genetic observations are relevant to the wider phenotype architecture because circulating SHBG is also associated with metabolic and cardiovascular outcomes. In postmenopausal women, higher SHBG has been associated with lower risk of type 2 diabetes [47], and genetic evidence involving *SHBG* rs1799941 has supported an inverse relationship between SHBG and type 2 diabetes susceptibility [48]. More recently, prospective and Mendelian randomisation analyses have also linked higher SHBG with lower coronary heart disease risk in women and men [49]. Against this background, the recurrence of SHBG, testosterone, adiposity, lipid, glycaemic, and cardiovascular phenotypes across different variants in the present study provides biologically coherent evidence of cross-domain connectivity. However, these relationships should not be interpreted as demonstrating that alteration of one measured hormone mediates the broader clinical associations, particularly where the same variant is associated with several correlated metabolic traits.

The *F2* rs1799963 findings provide a useful example of this distinction. The variant showed strong VTE susceptibility in FinnGen, but its broader phenotype profile was not confined to thrombosis: the strongest Pan-UKBB association was with triglycerides, while an FDR-supported association with SHBG provided an additional hormone-related connection. The latter is notable in the context of established genetic and epidemiological relationships between SHBG and metabolic phenotypes [11,47,48,49], but the substantially stronger triglyceride association argues against characterising *F2* primarily as a hormone-related locus in this dataset. Moreover, neither the SHBG association nor the nominal liver eQTL context establishes a pathway from *F2* through SHBG to thrombosis. Formal colocalisation was not performed, and the regional fine-mapping analysis itself showed incomplete resolution at *F2*, with rs1799963 sharing the 95% credible set with three correlated variants. This provides a concrete example of why phenotype association, molecular annotation, and locus resolution should be considered separately. The strong VTE association is nevertheless consistent with the established genetic architecture of venous thrombosis, in which prothrombin G20210A and Factor V Leiden represent major inherited susceptibility variants alongside a broader polygenic component [14].

The clearest clinically interpretable example emerged from the comparison of *F5* rs6025 and *F2* rs1799963. Both variants were strongly associated with VTE in the same FinnGen endpoint, but regional fine-mapping distinguished markedly different levels of statistical resolution: rs6025 accounted for almost all posterior probability at the *F5* locus and alone constituted its 95% credible set, whereas rs1799963 remained within a four-variant credible set at *F2*. The importance of these loci for the menopause context derives not from the discovery of their thrombotic effects, which are well established, but from the clinical relevance of inherited thrombotic susceptibility when exogenous oestrogen exposure is considered. Factor V Leiden and prothrombin G20210A have both been associated with increased VTE risk, and several studies have specifically examined their relationship with postmenopausal hormone exposure [24,25,50,51]. In the ESTHER study, Straczek et al. found that inherited Factor V Leiden or prothrombin G20210A and oral oestrogen exposure jointly identified women with substantially increased VTE risk, while the corresponding observational estimates for transdermal oestrogen did not indicate an additional increase beyond that associated with the prothrombotic mutation itself [25]. Other observational studies and meta-analyses have similarly reported higher VTE risk with oral than transdermal oestrogen and variation according to hormone formulation [21,22,23,52]. Randomised evidence has also established increased VTE risk with oral oestrogen-plus-progestin regimens in relevant postmenopausal populations [53]. Current menopause guidance consequently emphasises that MHT risk varies according to factors including age, timing, formulation, dose, route, and individual clinical risk [9,15,16]. The present results should therefore be interpreted as establishing inherited VTE susceptibility and differences in locus resolution, while the external clinical literature establishes why such susceptibility may be relevant to hormone exposure. They do not constitute a genotype-by-MHT interaction analysis and do not demonstrate that rs6025 or rs1799963 should independently determine MHT selection, formulation, dose, or route.

More broadly, the observed heterogeneity has implications for how germline information might eventually contribute to genetic susceptibility assessment around menopause. Previous work has investigated genetic prediction of early menopause and ANM-associated health outcomes, ranging from combinations of established menopause-associated variants to contemporary polygenic approaches [1,26,27,54]. The present study addresses a different level of information. Rather than deriving a polygenic risk score or predicting menopause timing, it shows that individual menopause-relevant variants may differ substantially in phenotype specificity, cross-domain breadth, statistical consistency, genetic independence, and locus resolution. These properties could ultimately be relevant when genetic findings are contextualised alongside conventional clinical susceptibility factors, particularly where the same variant is connected to reproductive, hormonal, metabolic, or clinically consequential phenotypes. However, association architecture alone is not equivalent to prediction. Clinical implementation of genomic susceptibility measures requires explicit evaluation of discrimination, calibration, incremental predictive value, generalisability, and clinical utility, and the performance of genetic risk models may differ substantially between populations [55,56,57]. Accordingly, the current findings support further evaluation of germline susceptibility information rather than the immediate construction of a diagnostic or treatment-selection framework.

Translation into individual-level menopause care would require a substantially different study design. Prospective cohorts would be needed to determine whether the cross-phenotype information identified here improves prediction beyond established clinical factors, with predefined outcomes, individual-level genotype and exposure information, appropriate calibration and discrimination analyses, and external validation in independent and ancestrally diverse populations [55,56,57]. Treatment-specific applications would require an additional evidentiary step: direct genotype-by-treatment analyses incorporating MHT exposure, formulation, route, dose, timing of initiation, duration, and baseline clinical risk. This is particularly important because contemporary recommendations already individualise MHT according to symptom indication, age and time since menopause, contraindications and comorbidities, and treatment characteristics including route and formulation [9,15,16]. The established differences in thrombotic risk between oral and transdermal oestrogen further demonstrate why genetic associations with VTE cannot simply be translated into treatment recommendations without exposure-specific evidence [21,23,25,52]. Future studies should therefore test whether genomic information provides clinically meaningful incremental information within these existing decision frameworks, rather than assuming clinical utility from statistical association alone.

Several features strengthen the present analysis. The study combined complementary genomic resources while preserving them as distinct evidence layers, incorporated both association strength and between-study heterogeneity, evaluated population-scale phenotypic associations with explicit multiple-testing correction, examined a prespecified subset of clinically relevant outcomes, and integrated disease-specific VTE evidence with LD and regional fine-mapping. The separation of association, heterogeneity, LD, fine-mapping, and molecular annotation also avoided reducing biologically different forms of evidence to a single composite score. Nevertheless, several limitations define the appropriate interpretation. First, the 31 variants were prespecified from an existing menopause-relevant evidence base and therefore do not represent an unbiased genome-wide discovery set. Second, the contributing summary-statistic resources may contain overlapping participants, and phenotype definitions, study numbers, and analytical designs differed across evidence sources; the marked I^2^ observed for several associations illustrates the importance of this heterogeneity. Third, the major genomic resources were predominantly derived from populations of European ancestry, limiting generalisability and potentially affecting both association and LD-based inference, an established concern in the translation of genetic risk information across populations [56,58]. Fourth, reliance on summary statistics precluded individual-level prediction, calibration, detailed covariate modelling, and direct genotype-by-treatment analyses. Fifth, the targeted PheWAS was restricted to predefined endpoints and should not be interpreted as an exhaustive phenome-wide discovery analysis. Finally, regional ABF fine-mapping assumed a single causal signal, while eQTL overlap and other molecular annotations were not equivalent to formal colocalisation or experimental evidence of mechanism. Collectively, these findings indicate that menopause-relevant germline variation is characterised not by a single dimension of genetic evidence, but by heterogeneous combinations of phenotypic breadth, statistical consistency, clinical association, genetic independence, and locus resolution. Recognising these distinctions may provide a more rigorous basis for future evaluation of genetic susceptibility around menopause, while prospective, individual-level, and treatment-specific studies remain necessary before such information may be translated into individual risk prediction or therapeutic decision-making.

## 5. Conclusions

This study demonstrates that menopause-relevant germline variants exhibit heterogeneous cross-phenotype architectures spanning reproductive and hormonal traits as well as metabolic, cardiovascular, skeletal, and thrombotic phenotypes. Importantly, association strength, between-study consistency, phenotypic breadth, genetic independence, and locus resolution were not interchangeable properties of the evidence. By integrating these dimensions at the individual-variant level, rather than focusing exclusively on menopause timing or isolated phenotypes, the present study provides a variant-centred framework for characterising the broader architecture of inherited susceptibility relevant to menopause. The contrasting evidence observed across hormone-related loci and clinically relevant variants, particularly the different cross-phenotype and locus-resolution profiles of *F5* and *F2*, further illustrates why strong genetic association alone does not imply equivalent biological or clinical interpretation.

These findings provide a basis for investigating how cross-phenotype germline information might contribute to future assessment of inherited susceptibility in the context of menopause and hormone exposure. Translation to individual risk prediction, clinical decision-making, or treatment selection will require prospective validation using individual-level clinical and exposure data, independent populations, and direct evaluation of genotype–treatment interactions.

## Figures and Tables

**Figure 1:**
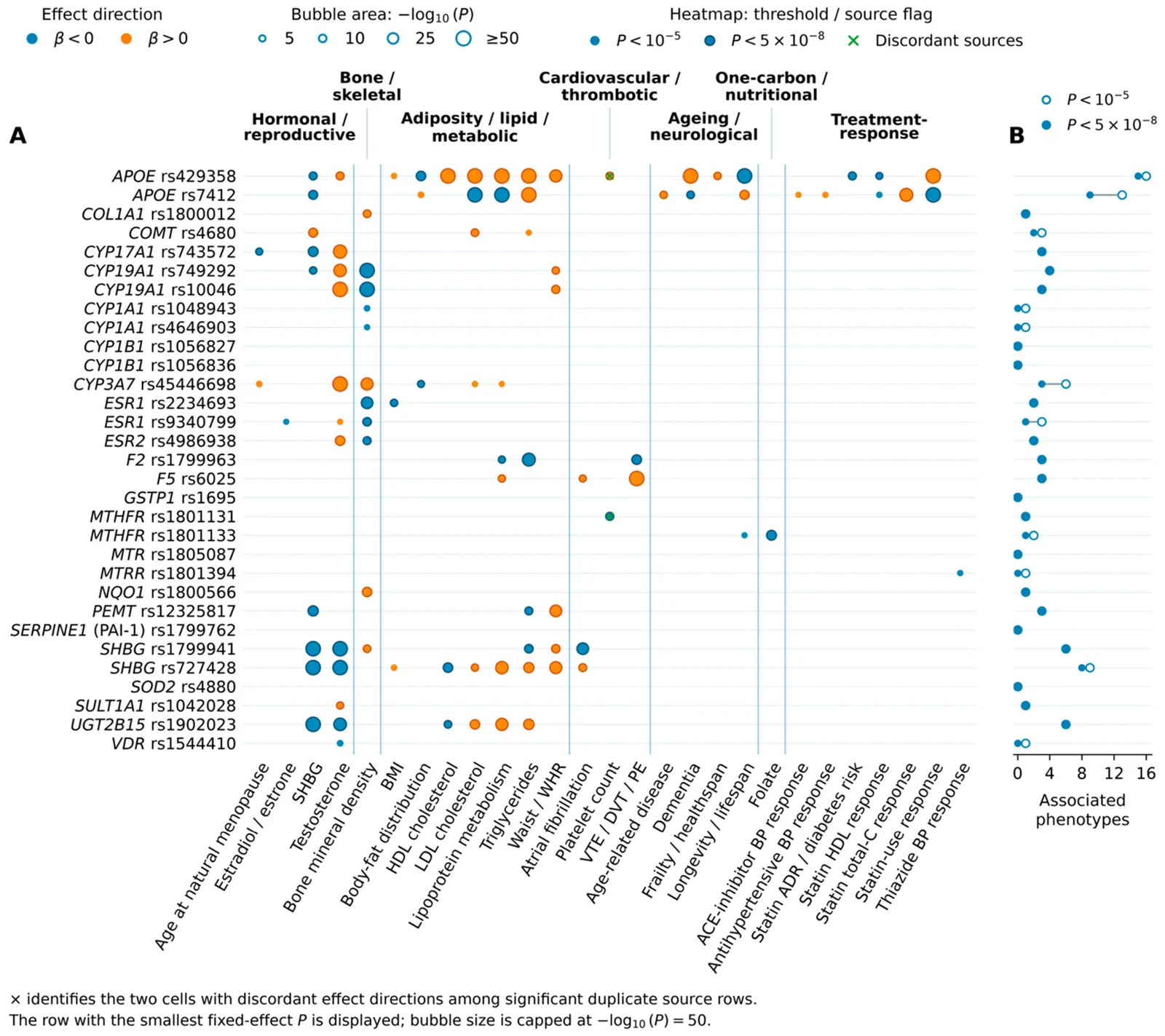
**Local cross-trait association landscape across prespecified menopause-relevant germline variants.** (**A**) Bubble matrix showing 95 unique variant–phenotype associations reaching *p* < 1 × 10^−5^ in the local cross-trait analysis, involving 25 of the 31 prespecified variants and 27 phenotypes. All 31 variants are retained as rows to preserve the complete prespecified variant set, including variants without associations meeting the displayed threshold. Phenotypes are arranged into broad trait groups for visual organisation only. Bubble size represents fixed-effect association strength as −log_10_(*p*), capped at 50 for visualisation of extremely small *p* values, while colour indicates the direction of the fixed-effect β. Associations reaching genome-wide significance (*p* < 5 × 10^−8^) are distinguished by a heavier outline. Blank cells indicate that no association meeting *p* < 1 × 10^−5^ was retained for the corresponding variant–phenotype pair. Where multiple significant source records mapped to the same variant–phenotype combination, a single association was retained for visualisation using the smallest fixed-effect *p* value. (**B**) Variant-level cross-phenotype breadth, expressed as the number of unique associated phenotypes per variant at *p* < 1 × 10^−5^ and *p* < 5 × 10^−8^. Overall, 25 of 31 variants were associated with at least one phenotype at *p* < 1 × 10^−5^ and 21 of 31 at least one phenotype at genome-wide significance. Phenotypic breadth ranged from 1 to 16 among variants with associations at *p* < 1 × 10^−5^ and from 1 to 15 among variants with genome-wide-significant associations. Trait grouping is provided for visual organisation and does not represent an additional statistical classification or independent evidence layer.

**Figure 2:**
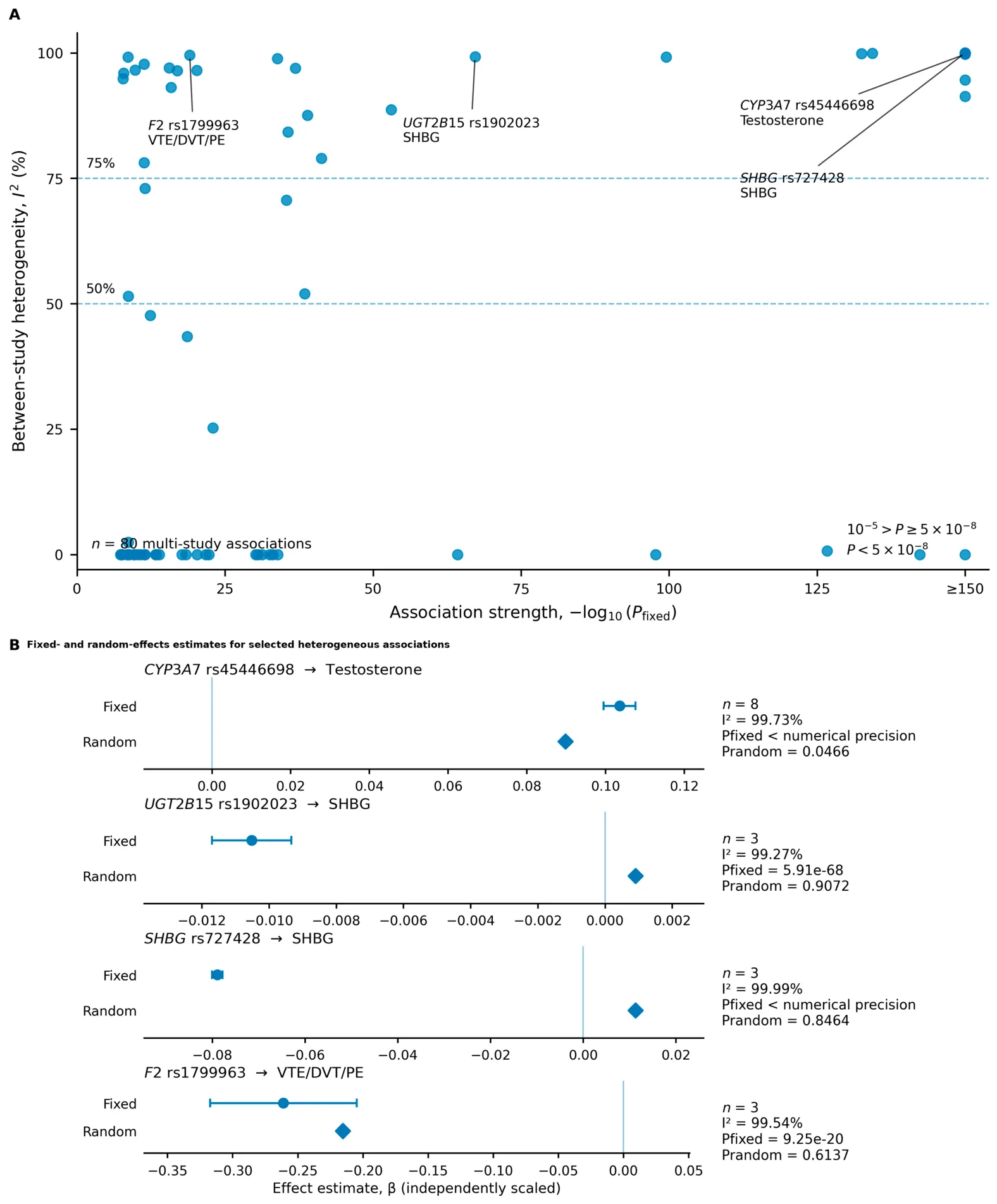
**Association strength and between-study heterogeneity among menopause-relevant cross-trait associations.** (**A**) Relationship between fixed-effect association strength and between-study heterogeneity for the 80 associations supported by at least two contributing studies and reaching *p* < 1 × 10^−5^ in the local cross-trait meta-analysis. Association strength is expressed as −log_10_(*p*_fixed), with values exceeding the plotting range displayed at ≥150; *p* values below numerical precision were assigned to this upper plotting boundary and were not interpreted as *p* = 0. Filled circles represent the associations included in the analysis. Horizontal lines at I^2^ = 50% and 75% are provided as reference guides for increasing between-study heterogeneity rather than categorical evidence thresholds. Overall, 38/80 (47.5%) associations had I^2^ > 50% and 32/80 (40.0%) had I^2^ > 75%. Selected highly heterogeneous associations are labelled to illustrate that strong statistical significance may coexist with substantial between-study variability. (**B**) Comparison of fixed- and random-effects estimates for four selected associations with high heterogeneity: CYP3A7 rs45446698 with testosterone, UGT2B15 rs1902023 with SHBG, SHBG rs727428 with SHBG, and *F2* rs1799963 with VTE/DVT/PE. Fixed-effect estimates are shown with 95% confidence intervals calculated from the corresponding fixed-effect standard errors, whereas random-effects estimates are shown as point estimates because random-effects standard errors or confidence intervals were not available in the source data. Effect-estimate axes are independently scaled for each association and are intended for within-association comparison only. The number of contributing studies (*n*), I^2^, and fixed- and random-effects *p* values are reported alongside each comparison. Together, the panels illustrate that association strength and between-study consistency represent distinct properties of the genetic evidence.

**Figure 3:**
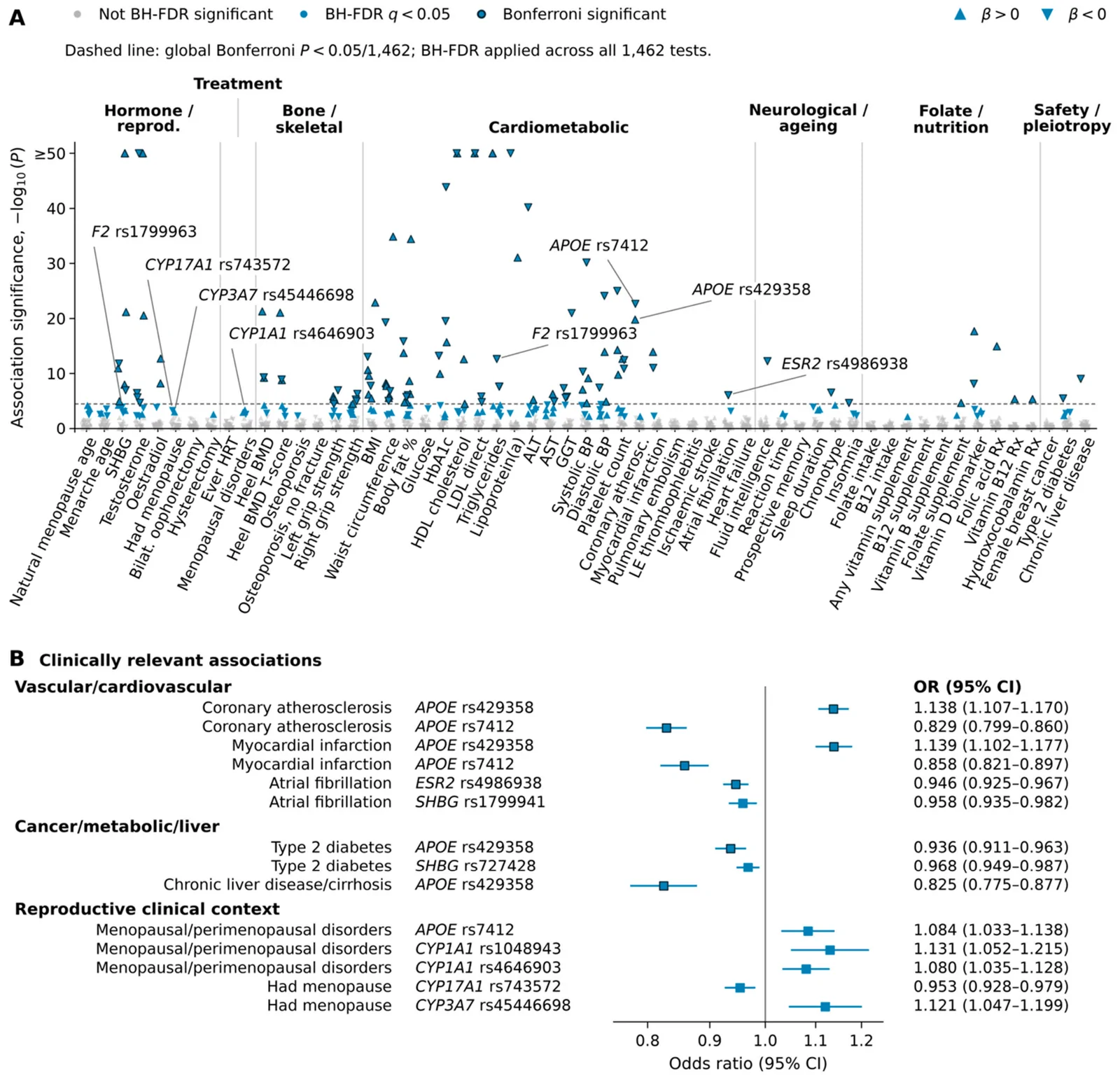
**Targeted Pan-UK Biobank phenome-wide association analysis and clinically relevant cross-phenotype associations.** (**A**) Phenome-wide association plot of 1462 variant–phenotype tests comprising 28 represented variants and 57 endpoints, arranged across seven predefined endpoint families. Each point represents one variant–endpoint association, with the *y*-axis showing association significance as −log_10_(*p*). Upward and downward triangles indicate positive and negative β estimates, respectively. Associations meeting Benjamini–Hochberg false discovery rate correction (BH-FDR q < 0.05) are distinguished from associations not meeting the FDR criterion, while associations additionally exceeding the Bonferroni threshold (*p* < 0.05/1462) are indicated by a heavier outline. The dashed horizontal line denotes the Bonferroni significance threshold. No horizontal FDR threshold is shown because BH-FDR significance was determined from the ranked set of tests rather than from a universal raw-P cut-off. For visualisation, −log_10_(*p*) values exceeding 50 are displayed at the upper plotting boundary. Selected corrected associations are labelled to illustrate reproductive/hormonal, cardiometabolic, and cardiovascular signals; all associations remain represented irrespective of labelling. Endpoint families are used for visual organisation of the targeted PheWAS and do not represent additional statistical classifications. (**B**) Effect-size representation of the 14 associations within the prespecified clinically relevant outcome subset that met BH-FDR q < 0.05. Squares indicate odds ratios (ORs) and horizontal lines their 95% confidence intervals, with the vertical reference line at OR = 1. Associations are grouped according to their clinical outcome domain. Associations additionally meeting the Bonferroni criterion are indicated by a black-outlined square. Effect estimates correspond to the analysed alternate allele and should be interpreted as association estimates rather than measures of individual-level risk or treatment response.

**Figure 4:**
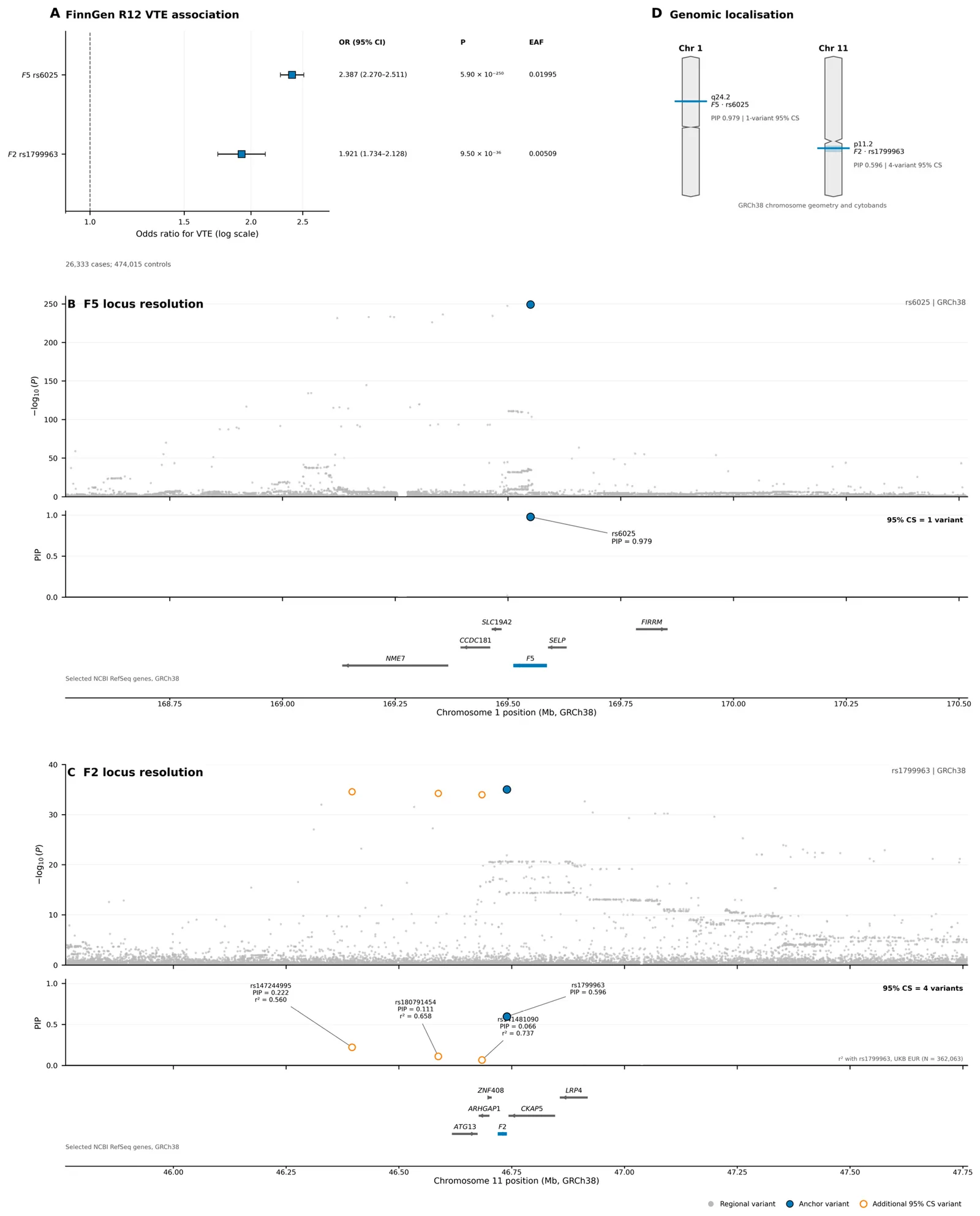
***F5* and *F2* venous thromboembolism susceptibility and locus resolution.** (**A**) FinnGen R12 associations of *F5* rs6025 and *F2* rs1799963 with venous thromboembolism (VTE), based on 26,333 cases and 474,015 controls. Squares indicate odds ratios (ORs) and horizontal lines indicate 95% confidence intervals; effect-allele frequencies (EAFs) and association *p* values are shown alongside. (**B**) Regional association and fine-mapping results for the *F5* locus. The upper track shows FinnGen VTE association strength across the analysed GRCh38 region as −log_10_(*p*), with rs6025 highlighted. The posterior inclusion probability (PIP) track shows the Wakefield approximate Bayes factor fine-mapping results; rs6025 had PIP = 0.979 and constituted the single-variant 95% credible set. Selected neighbouring genes are shown using GRCh38 genomic coordinates. (**C**) Corresponding regional association and fine-mapping results for the *F2* locus. The 95% credible set comprised four variants: rs1799963 (PIP = 0.596), rs147244995 (PIP = 0.222), rs180791454 (PIP = 0.111), and rs141481090 (PIP = 0.066). For the three non-anchor credible-set variants, r^2^ with rs1799963 is shown using the UK Biobank European LD reference (*N* = 362,063). LD information is provided as an annotation and was not used in the ABF calculation. (**D**) GRCh38 genomic localisation of the *F5* rs6025 locus on chromosome 1q24.2 and the *F2* rs1799963 locus on chromosome 11p11.2, together with their lead-variant PIPs and 95% credible-set sizes. Grey points represent regional variants, blue filled points indicate the anchor variants, and orange outlined points indicate additional variants belonging to the 95% credible set. CS, credible set; EAF, effect-allele frequency; LD, linkage disequilibrium; OR, odds ratio; PIP, posterior inclusion probability; VTE, venous thromboembolism.

**Table 1:** **Prespecified germline variants included in the cross-phenotype analysis.** The 31 variants represent a literature-curated set selected a priori on the basis of previously reported biological or genetic relevance to menopause-associated physiology and clinically relevant susceptibility, including sex-steroid synthesis, metabolism, transport and signalling, reproductive ageing, skeletal biology, one-carbon metabolism, oxidative metabolism, cardiometabolic traits, and thrombotic susceptibility. Variant selection was intended to capture biologically relevant germline variation across complementary aspects of menopause-related physiology rather than to constitute a validated diagnostic or predictive panel. Gene/locus assignments, GRCh37 genomic coordinates, and allele information were harmonised during data curation. The rationale column summarises the original biological basis for inclusion and is independent of the cross-phenotype association, heterogeneity, linkage-disequilibrium, and fine-mapping results generated in the present study.

Gene/Locus	rsID	Chr:Position (GRCh37)	Effect/Reference Alleles	Original Menopause-Relevant Rationale
***APOE***	rs429358	19:45,411,941	C/T	Lipid transport and cardiometabolic, vascular, and neurovascular susceptibility
***APOE***	rs7412	19:45,412,079	T/C	Lipid transport and cardiometabolic, vascular, and neurovascular susceptibility
***COL1A1***	rs1800012	17:48,277,749	A/C	Type I collagen biology and skeletal/bone-remodelling susceptibility
***COMT***	rs4680	22:19,951,271	A/G	Catechol-oestrogen methylation and oestrogen-metabolite clearance
***CYP17A1***	rs743572	10:104,597,152	G/A	Steroidogenesis and sex-steroid biosynthesis
***CYP19A1***	rs10046	15:51,502,986	A/G	Aromatase activity and oestrogen biosynthesis
***CYP19A1***	rs749292	15:51,558,731	A/G	Aromatase activity and oestrogen biosynthesis
***CYP1A1***	rs1048943	15:75,012,985	C/T	Oxidative oestrogen metabolism and hydroxylation
***CYP1A1***	rs4646903	15:75,011,641	G/A	Oxidative oestrogen metabolism and hydroxylation
***CYP1B1***	rs1056827	2:38,302,177	A/C	Oxidative oestrogen metabolism and catechol-oestrogen formation
***CYP1B1***	rs1056836	2:38,298,203	G/C	Oxidative oestrogen metabolism and catechol-oestrogen formation
***CYP3A7***	rs45446698	7:99,332,948	G/T	Steroid-hormone metabolism and circulating sex-steroid regulation
***ESR1***	rs2234693	6:152,163,335	C/T	Oestrogen-receptor-α signalling and tissue response to oestrogens
***ESR1***	rs9340799	6:152,163,381	G/A	Oestrogen-receptor-α signalling and tissue response to oestrogens
***ESR2***	rs4986938	14:64,699,816	T/C	Oestrogen-receptor-β signalling and tissue response to oestrogens
***F2***	rs1799963	11:46,761,055	A/G	Prothrombin biology and inherited venous-thrombotic susceptibility
***F5***	rs6025	1:169,519,049	C/T	Factor V biology and inherited venous-thrombotic susceptibility
***GSTP1***	rs1695	11:67,352,689	G/A	Glutathione-dependent detoxification of reactive metabolites and oxidative-stress products
***MTHFR***	rs1801131	1:11,854,476	G/T	Folate-dependent one-carbon metabolism and homocysteine regulation
***MTHFR***	rs1801133	1:11,856,378	A/G	Folate-dependent one-carbon metabolism and homocysteine regulation
***MTR***	rs1805087	1:237,048,500	G/A	Methionine synthesis, folate-dependent one-carbon metabolism, and homocysteine regulation
***MTRR***	rs1801394	5:7,870,973	G/A	Methionine-synthase regeneration, one-carbon metabolism, and homocysteine regulation
***NQO1***	rs1800566	16:69,745,145	A/G	Quinone detoxification and oxidative metabolism of oestrogen-derived metabolites
***PEMT***	rs12325817	17:17,486,519	G/C	Hepatic phosphatidylcholine synthesis and choline-dependent one-carbon biology
***SERPINE1 (PAI-1)***	rs1799762	7:100,769,707	4G/5G	Regulation of fibrinolysis through plasminogen activator inhibitor-1
***SHBG***	rs1799941	17:7,533,423	A/G	Sex hormone-binding globulin concentrations and circulating sex-hormone bioavailability
***SHBG***	rs727428	17:7,537,792	C/T	Sex hormone-binding globulin concentrations and circulating sex-hormone bioavailability
***SOD2***	rs4880	6:160,113,872	G/A	Mitochondrial antioxidant defence and oxidative-stress biology
***SULT1A1***	rs1042028	16:28,617,514	T/C	Sulfation and conjugative clearance of oestrogens and catechol-oestrogen metabolites
***UGT2B15***	rs1902023	4:69,536,084	C/A	Glucuronidation and clearance of steroid hormones
***VDR***	rs1544410	12:48,239,835	T/C	Vitamin D receptor signalling and skeletal/bone-remodelling biology

## Data Availability

The principal results are presented within the article. Additional supporting data, including the complete variant–trait association tables, targeted PheWAS results, and regional fine-mapping outputs, are available from the corresponding author upon reasonable request.
